# A novel digital tool to guide provision of near vision glasses for presbyopia correction in the community

**DOI:** 10.3389/fopht.2026.1891054

**Published:** 2026-08-10

**Authors:** Marzieh Katibeh, Shalinder Sabherwal, Mohd Javed, Elanor Watts, Sergio Latorre-Arteaga, Nigel M. Bolster, Darren Coverley, Alice Pintus, Vince Hewitt, Nam Thaker, Andrew Bastawrous

**Affiliations:** 1 Peek Vision, London, United Kingdom; 2 Department of Community Ophthalmology and Public Health Research, Dr Shroff’s Charity Eye Hospital, New Delhi, India; 3 International Centre for Eye Health, London School of Hygiene and Tropical Medicine, London, United Kingdom; 4 Tennent Institute of Ophthalmology, Glasgow, United Kingdom; 5 Public Health Research Group, University of Alicante, Alicante, Spain

**Keywords:** digital health, effective refractive error coverage, glasses, mHealth, mobile health, near vision, presbyopia, spectacles

## Abstract

**Purpose:**

To develop and validate a digital tool to support non-eye specialists, such as community health workers (CHWs), to provide appropriate near vision glasses to correct presbyopia, in line with the WHO SPECS 2030 initiative.

**Methods:**

Epidemiological data, and expert survey results, were combined to inform design of a digital tool for the correction of presbyopia. This validity study (a cross-sectional/methods comparison study) took place in Uttar Pradesh, in Northern India, with 378 participants with uncorrected distance visual acuity 6/12 or better in both eyes, but near visual impairment. The difference between near correction power (diopters) recommended by use of the Peek presbyopia calculator in the hands of CHWs at the household, and by optometrist (gold standard) using standard methods in a clinic, was compared using Bland-Altman analysis.

**Results:**

Powers of near vision glasses, as suggested by the Peek presbyopia calculator used by a community health worker, agreed exactly with optometrist-prescribed powers in 92.9% of cases, and were within 0.50D in 99.5% of cases. The mean bias was negligible (-0.012D, SD = 0.15, p=0.12), with a 95% limit of agreement between -0.301 and +0.277 diopters.

**Discussion:**

The newly developed Peek presbyopia calculator guided provision of near vision glasses for presbyopia with excellent agreement compared to optometrist recommendations, and can be used by non-eye specialists to provide appropriate ready-made near vision glasses for presbyopia correction.

## 1 Introduction

Approximately 1 billion people globally have avoidable vision impairment (VI). Uncorrected presbyopia - age-related loss of accommodation - is by far the most common cause, responsible for an estimated 826 million cases of near VI (1, 2). In the vast majority of cases, presbyopia can be easily and cheaply corrected with a pair of ready-made near vision spectacles, or “reading glasses”. Near vision glasses enable not only reading but all near tasks, and as such have been shown to improve quality of life and income (3). The proportion of people with refractive error or presbyopia whose VI has been appropriately corrected can be described using the effective refractive error coverage (eREC) metric. In 2021, global near vision eREC was estimated to be 20.5% (4). The 74th World Health Assembly endorsed global targets for eREC with a goal to increase eREC by 40%, from 36% to 76%, by 2030 (5). To reach this goal, the WHO launched the SPECS 2030 initiative (6). This initiative is structured around five pillars of refractive services: improving access, building capacity of personnel, improving population education, reducing cost, and strengthening surveillance and research.

Due to their simplicity and negligible risk, in many countries, including Australia, the UK and US, ready-made near vision glasses are available cheaply over the counter e.g. in pharmacists or supermarkets, and are considered low-risk, exempt from strict regulation. The WHO Summary Guide on Quality Standards for Spectacles recommends that ready-made near vision spectacles for presbyopia can be purchased without a prescription, and the WHO have recommended availability of near vision glasses over-the-counter and via primary and community healthcare workers (CHWs) (7, 8). A barrier to access to presbyopia correction is that in many settings this approach has not yet been successfully implemented: CHWs are not yet carrying out screening for presbyopia or providing correction, and may not have the training, confidence or enabling regulation to do so. Additional barriers include weak supply chains, limited availability in rural areas, and sociocultural perceptions of poor vision as a normal part of ageing (8).

In South Asia and Southeast Asia, there are a median of 10 and 5 optometrists per million people respectively (9). This figure drops to 2 per million in Sub-Saharan Africa, compared to 156 optometrists per million in high-income countries (9). Given that objective presbyopia is a universal condition among older adults, the need far outstrips that which can be provided by the optometry workforce. As such, there is a need for non-eye care specialists to accurately and confidently provide presbyopia correction.

The development of a digital near vision test embedded in the Peek eye health platform has previously been described (10). Where there is sufficient public understanding of near vision glasses, a preferred power of correction might then be self-selected by users. However where awareness is low, there is a need for a tool to support non-eye-specialists, e.g. community health workers, to aid selection of appropriate presbyopia correction. This is encouraged by the WHO Competency-based Refractive Error Teams framework (11), and could support pillars 1, 2, 4 and 5 of SPECS 2030, by cost-effectively expanding the workforce who are able to correct presbyopia, and allowing simultaneous data collection.

Although digital tools for measuring near visual acuity have been validated, including in our prior work, there remains a gap in tools that support the safe and consistent provision of near vision correction (9). In this study, we describe the development and validation of a new tool, the Peek presbyopia calculator, a decision-support tool designed to guide non-eye specialists in selecting appropriate near vision spectacles for people with presbyopia. Unlike earlier tools focused on measurement, this algorithm-driven calculator supports the prescription and dispensing process in community settings, enabling more accurate and scalable provision of presbyopia correction.

## 2 Materials and methods

### 2.1 Review of current practice

Firstly, available guides to presbyopia correction were reviewed, including: the WHO AFRO Primary Eye Care Training Manual (12), the Vision and Eye Screening Implementation Handbook (13), Summary Guide on Quality Standards for Spectacles (7), the Eye Care Competency Framework (14), Competency-based Refractive Error Team resources (11), and Community Eye Health Journal publications and training materials used in low- and middle-income settings (15). Commonly, age or presenting near visual acuity (NVA) were used to propose a likely dioptric power of near correction (16, 17). In previously published data of near vision correction requirements across nine countries, both age and uncorrected NVA indeed correlated with required power, with age as the strongest predictor (18).

Between November 2023 and January 2024, 16 optometrists and ophthalmologists with global eye health expertise, from countries including Kenya, Nepal, India, Spain, Iran, Australia, Mozambique and the UK, were surveyed regarding improving presbyopia correction in low- and middle-income countries (19). In this survey, participants completed a structured online questionnaire (see **Supplementary Material**) developed by the study team, comprising a mix of closed and open-ended questions covering current practices, factors influencing presbyopia correction, and potential improvements in service delivery. The survey was piloted internally first, to ensure clarity, consistency, and content validity.

Some key findings included:

- The preferred type of presbyopia correction for a low-income, community setting was ready-made single vision spectacles: “ready readers”.
- The most commonly recommended ages at which to start presbyopia screening and correction were 35 and 40 years.
- Only 1 respondent answered that there wasn’t a need to update provision of presbyopia correction.
- Qualitative results included recommendations to develop a decision aid tool which could support non-eye care workers to appropriately provide ready-made near vision glasses.

### 2.2 Tool design

The above review, epidemiological data, and expert survey results were combined to inform design of the new Peek presbyopia calculator tool. Peek is a widely used digital platform for eye health that supports screening, data capture, and analysis. The presbyopia calculator is a decision-support tool integrated into Peek’s broader system for use in general eye health programs.

It is delivered via smartphones or tablets, intended to help non-eye specialists provide ready-made near vision spectacles safely and consistently in community settings.

For patients aged 35 or over, following collection of demographic data, screening questions, and monocular distance acuity testing, NVA is tested via the app, as part of the screening process. This is done binocularly using Tumbling E optotypes, via a previously validated digital NVA test (10). The calculator uses a data-derived algorithm derived from large program datasets (over 33, 000 participants (18)) to recommend an initial near-add starting point using three inputs collected at the point of care: age, uncorrected near visual acuity (NVA), and prior cataract surgical history. This suggests a power of near vision correction (diopters) that the person is likely to require, from the range: +1.00D, to +3.50D at 0.50D intervals.

**Figure 1:**
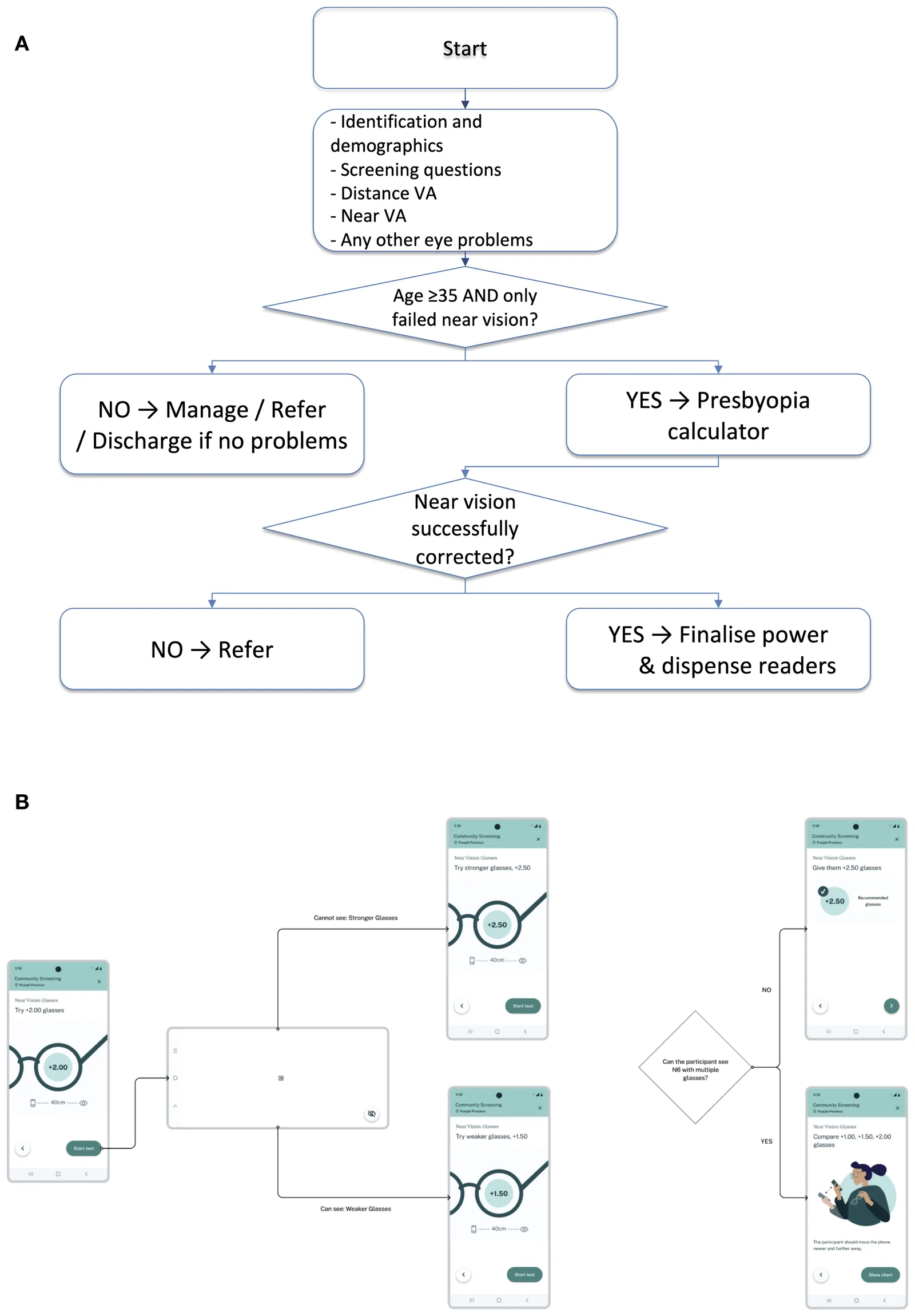
Flowchart of the screening and presbyopia correction workflow. **(a)**: The decision pathway used to identify individuals eligible for the presbyopia calculator based on age and screening results (distance and near visual acuity and other eye conditions). Individuals meeting the criteria proceed to the presbyopia calculator, while others are managed or referred as appropriate. **(b)**: The app-based algorithm, which guides users through iterative selection of near-vision lens power. Based on NVA and user feedback (ability to see with different lens strengths), the app refines the recommendation until an appropriate near correction is identified and dispensed, or referral is indicated.

The participant wears this suggested power of near vision glasses and re-tests ability to see N6. From that initial recommendation, the tool then runs a guided, structured refinement loop: the user is guided through repeat near-vision screening and stepwise trial of alternative diopters until near vision impairment is corrected. These are trialed by the patient in the form of pairs of ready-made near vision spectacles. NVA is re-tested to assess whether the near vision impairment (NVI) has been corrected: i.e. whether the patient can now see N6 at 40cm. If more than one power produces an acceptable functional result, the participant is prompted to compare options and choose the most comfortable (user preference step). If the patient’s NVI cannot be corrected with ready-made near vision spectacles via this process, or if any other visual/eye concerns have been raised during the screening process, they are referred onwards for more detailed assessment. See **Figure 1**. This digital tool is not intended to be a substitute for comprehensive refractive or ophthalmological examination.

### 2.3 User feedback and iterative improvement

Following the initial development process, the tool was piloted with 32 patients and three testing teams, and interviews were conducted with screeners, clinicians and patients involved in the initial piloting. During pilot testing, agreement between the screener-administered presbyopia calculator and the optometrist’s recommendation was excellent (Lin’s Concordance Correlation Coefficient 0.911, 95% CI: 0.829–0.955) and the four discordant cases were distributed across all three screeners (1:1:2). Feedback from the users was then used to optimize the test design, flow and usability: via addition of explanatory illustrations, educational screens, and allowing patients to trial glasses at their preferred working distance. Representative images of participants involved in the study are provided in the **Supplementary Material**, with consent to share.

### 2.4 Objective

To assess the accuracy and reliability of the near vision correction prescription provided by a screener using the Peek presbyopia calculator, versus the gold standard (assessment and provision of presbyopia correction by optometrist).

### 2.5 Study design and participants

This is a cross-sectional observational validity study. The study setting was the catchment of Dr Shroff’s Charity Eye Hospital in Khiri district, Uttar Pradesh, in Northern India. A simple random cluster method was used.

Sample size was calculated using Bland-Altman Limits of Agreement (20), using MedCalc (version 19.6.0, MedCalc Software, Ostend, Belgium). A minimum sample size of 271 was calculated, based on an α of 5%, 90% power, 95% limits of agreement, 0.10 expected standard deviation of difference, and 0.2 maximum tolerable difference.

The study’s inclusion criteria were adults aged ≥35 years, confirmed via age identification documents, with monocular uncorrected distance VA better than or equal to 6/12 in both eyes, binocular near VA worse than N6 at 40cm (equivalent to 0.27 LogMAR) i.e. NVI, who were willing to give consent and participate in the study. Corresponding exclusion criteria were individuals aged less than 35 years, monocular distance VA worse than 6/12 in either eye, binocular near VA N6 or better at 40cm, declining to participate, or those unable to give informed consent.

### 2.6 Data collection and management

Data was collected using Epicollect5 (Centre for Genomic Pathogen Surveillance, Oxford, UK) and the Peek digital near vision test and presbyopia calculator (Peek Vision, London, UK).

Data was exported in csv and ms-excel format. Data and all appropriate documentation will be stored for a minimum of 5 years after the completion of the study, including the follow-up period. Data were recorded digitally within password-protected devices, and paper records (including consent forms) were stored in locked cabinets.

This study was carried out as part of an ongoing door-to-door screening program conducted using the Peek platform in villages of Khiri district. Three community health workers were trained to use the calculator and collect data.

Eligibility to take part was confirmed and consent obtained following explanation of the objectives of the study. Participants’ demographics were collected including age, gender, and glasses ownership status. DVA was tested for each eye. NVA was then tested, with NVI defined as inability to see N6 at 40cm, in line with WHO definitions (21). Participants with good distance vision (uncorrected DVA 6/12 or better in both eyes), but NVI, proceeded to assessment for provision of ready-made near vision glasses: (a) using the Peek presbyopia calculator, and (b) as per Optometrist assessment. All optometrist assessments were performed by a single optometrist, removing inter-observer variability. Data was collected by the community health workers, and prescriptions generated using the calculator were stored in the app. Study participants were then referred to a nearby primary eye care center, where an optometrist — masked to the screener’s prescription — performed refraction in a controlled clinic environment. The results of the optometrist’s refraction were then compared to the prescriptions made by the screeners using the calculator, treating optometrist recommendation as the gold standard.

### 2.7 Data analysis

Data analysis was conducted using R-Statistical software, version 4.2.2 (National Institutes of Health, Bethesda, MD), and Stata Version 14 (Stata Corp, TX). NVA was initially measured in N-units in line with ICD definitions, and all VA measurements were converted to LogMAR for analysis.

The difference between near correction power (diopters) recommended by use of the new Peek presbyopia calculator and by optometrist was compared using Bland-Altman analysis.

### 2.8 Ethical considerations

The study was approved by the Institutional Review Board of Dr Shroff’s Charity Eye Hospital: reference IRB/2024/MAY/02. All participants gave informed written (or thumbprint) consent to participate. The study was conducted in accordance with the tenets of the Declaration of Helsinki.

## 3 Results

378 people participated in the study; participant demographics are described in **Table 1**.

**Table 1:** Participant demographics.

Characteristics	n (%)
Gender	
Female	217 (57.41%)
Male	161 (42.59%)
Age	
Mean ± SD	45.7 ± 6.7
Minimum	35
Maximum	80
Glasses ownership status	
Already owned glasses	15 (3.96%)
No glasses	363 (96.03%)
Near VA	
N8	140 (37.04%)
N10	117 (30.95%)
N12.8	78 (20.63%)
N16	25 (6.61%)
N20	16 (4.23%)
N25.6	2 (0.53%)
Total	378 (100%)

**Figure 2:**
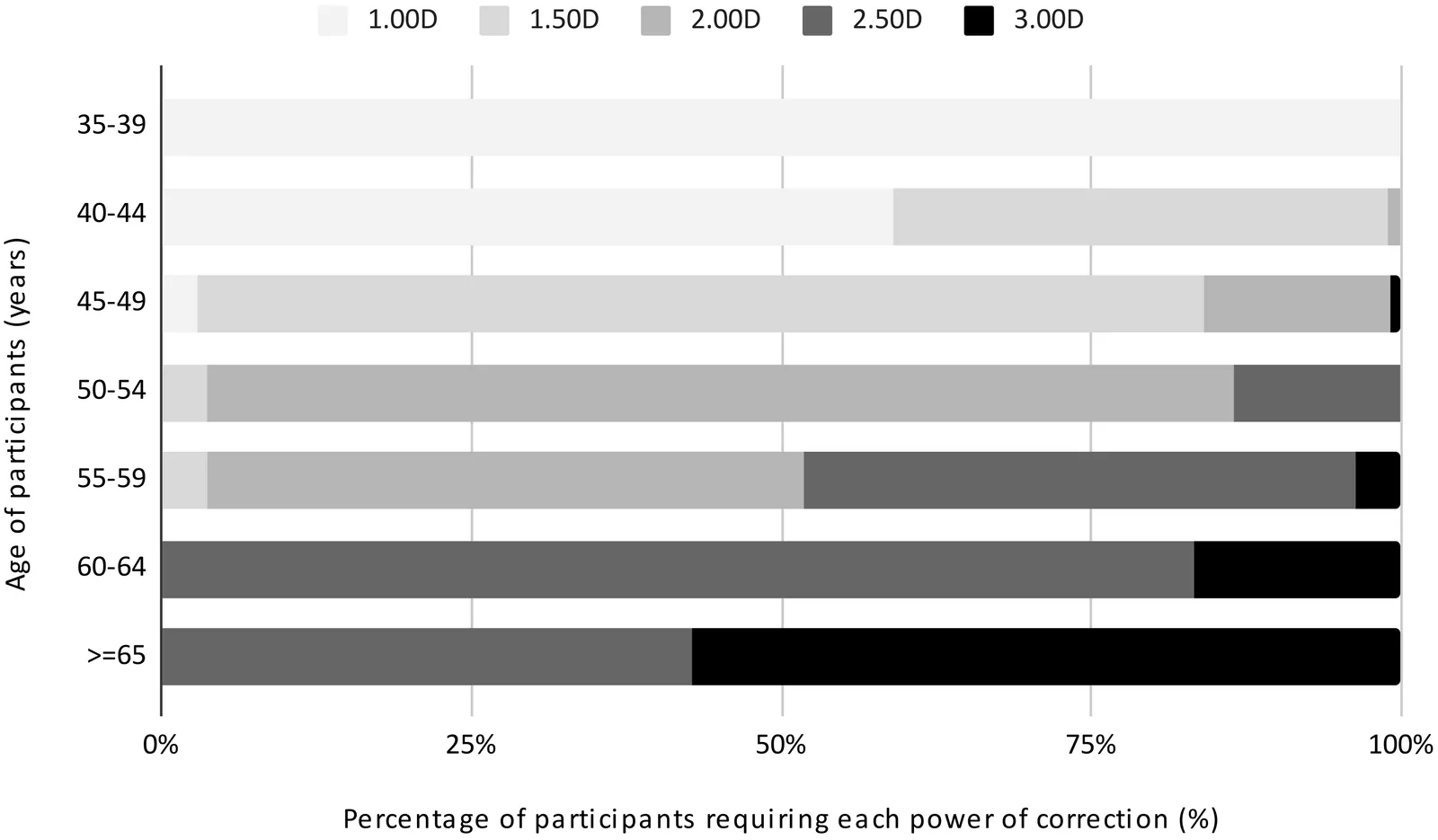
Distribution of required powers of near correction (diopters) according to optometrist assessment.

**Figure 2** depicts the distribution of required dioptric powers of near vision glasses according to optometrist assessment at the vision center, by age group, and **Table 2** shows the required powers according to assessment by both optometrist and Peek presbyopia calculator.

**Table 2:** Required powers of near correction (diopters) according to optometrist assessment and Peek presbyopia calculator.

Age (year)	Power (D) identified by optometrist					Power (D) by Peek presbyopia calculator				
	Mean	Median	SE	95% CI Lower	95% CI Upper	Mean	Median	SE	95% CI Lower	95% CI Upper
35-39	1	1	0	1	1	1	1	0	1	1
40-44	1.21	1	0.028	1.15	2.26	1.2	1	0.031	1.14	1.26
45-49	1.57	1.5	0.025	1.52	1.62	1.56	1.5	0.025	1.51	1.61
50-54	2.05	2	0.022	2	2.09	2.02	2	0.019	2	2.06
55-59	2.24	2	0.062	2.11	2.37	2.24	2	0.062	2.11	2.37
60-64	2.58	2.5	0.083	2.37	2.8	2.58	2.5	0.083	2.37	2.8
≥65	2.78	3	0.101	2.54	3.03	2.78	3	0.101	2.54	3.03

**Figure 3:**
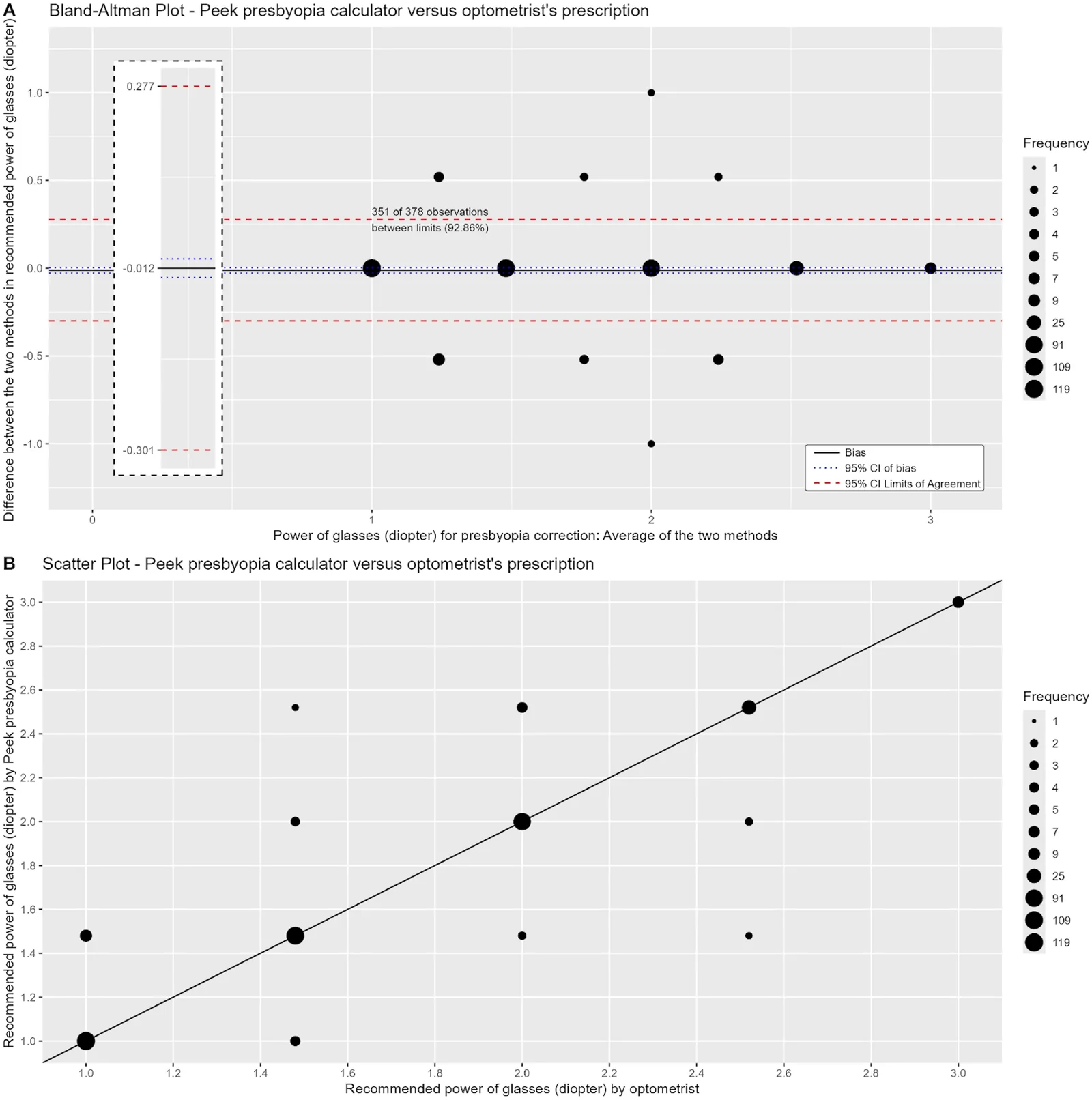
Comparison of power of near vision glasses (diopters) recommended by Peek presbyopia calculator vs optometrist: **(a)** Bland-Altman plot. The mean difference (bias) between recommended powers is indicated by the solid line, 95% CIs of this difference are indicated by dotted blue lines, and 95% limits of agreement are indicated by dotted red lines. **(b)** Scatter plot. Frequency describes the number of participants.

Powers of recommended near vision glasses, as suggested by the Peek presbyopia calculator used by a community health worker, agreed exactly with optometrist-prescribed powers in 92.9% of cases. Recommended powers were within 0.50D in 99.5%, with only two participants having a discrepancy of 1.00D, and none greater than this. The mean bias was negligible (-0.012), with a 95% limit of agreement between -0.301 and +0.277 diopters. See **Figure 3**.

The mean time taken to provide a presbyopia correction prescription using the Peek app: time between participant failing the near vision test and the final near vision prescription by Peek presbyopia calculator, was 137.45 seconds (CI 95%: 133.74-141.17).

## 4 Discussion

Presbyopia correction improves productivity (22), income (3), and empowerment (23), and is a low-cost, safe intervention. It should, therefore, be a “low-hanging fruit” within the spheres of eye care, healthcare and development. However, there remain hundreds of millions of people globally with near vision impairment due solely to lack of near vision glasses (1). The WHO SPECS 2030 Initiative aims to dramatically improve both near and distance effective refractive error coverage (eREC). Distance eREC often requires skilled eye care workers: optometrists or ophthalmologists to provide personalized refractive services. The majority of near correction required is simple presbyopic correction, which can be provided in the form of ready-made near vision glasses. These do not require a trained specialist, and there are not enough optometrists in many regions of the world to meet the global presbyopic need (9). The WHO Competency-based Refractive Error Teams (CRET) document specifically encourages the use of Introductory Level 1 cadres (including community health workers) to provide and dispense ready-made near vision spectacles for presbyopia (11). The Peek presbyopia calculator described in this article is designed to support non-eye care specialists, such as community health workers, pharmacists or entrepreneurs, who could provide near vision glasses, with the added guidance and confidence provided by such a tool. Some eye care providers have previously expressed concerns that a focus on presbyopia could cause other conditions to be overlooked, however as this tool is designed to be embedded within a wider screening and referral platform, people with other eye conditions would be referred accordingly. In addition, provision of presbyopia correction by non-specialists can reduce pressure on eye care services by freeing up scarce specialist resources to focus on conditions that require their skills, in line with the WHO CRET approach.

Our validity study found extremely high agreement between ready-made near vision glasses recommended by an experienced optometrist, and by a CHW using the Peek presbyopia calculator. The overall bias was −0.012 D, which is both statistically and clinically insignificant, and the Bland-Altman 95% limits of agreement were narrow (−0.301 to +0.277 D), i.e. within approximately ±0.3 D and centred close to zero. 351 of 378 participants (92.9%) showed exact agreement. This demonstrates that the Peek presbyopia calculator can act as a validated option to support the provision of near vision glasses by non-eye specialists. Screeners using the tool reported increased confidence, with a sense of being supervized, and reduced reluctance to provide reading glasses.

Study limitations include an age skew among the participants, who were relatively young presbyopes: mean age 45.7 years. The results may be less generalizable to older people. Similarly, as the study was carried out in India, the results may be less applicable to populations in other regions. Also, this validity study evaluated the calculator in adults with distance VA ≥6/12, reflecting its initial use-case: rapid selection of ready-made near spectacles where distance vision is not significantly impaired. For individuals with co-existing refractive error (i.e. myopia, hypermetropia or astigmatism), the current pathway is referral for bespoke refraction or further assessment; future development will explore how the tool can be integrated into broader service pathways and updated as additional program data accumulate. In this initial study, we compared to optometrist prescription to confirm whether the tool can be used to accurately provide presbyopia correction; however, we did not here compare to other field-based/CHW methods of provision and we assessed each screener’s agreement against the gold standard rather than inter-rater reliability directly. However, the even distribution of discordant cases across the three screeners suggests minimal between-tester variability.

There are other available methods and training available to support provision of presbyopia correction. These include the WHO Training in Assistive Products (TAP) Reading Glasses module (24). The method recommended in the TAP module to select appropriate correction is to start with a power of +1.00D for all relevant people, and then keep increasing in power until the person can see small optotypes clearly. Of note, there are planned updates for the WHO TAP Reading Glasses module, as it has previously featured an N8 threshold, which is not aligned with updated WHO/ICD definitions for near vision impairment (N6) (21). In addition, while the TAP approach is very sensible and should be encouraged, we hope that the Peek presbyopia calculator will allow identification of the right power more quickly, by starting with a more appropriate power, rather than always starting at +1.00D.

Another option for near vision glasses selection is self-selection, or trial and error by the person with presbyopia, without any healthcare worker involvement at all. This is also a good alternative option, especially in areas where there is already some understanding of presbyopia, and has been encouraged by WHO (8). The Peek presbyopia calculator however will be useful in regions where community awareness is not yet sufficient for people to spontaneously seek out correction, and so where guidance is required from a healthcare worker. When used as part of a more comprehensive community eye health program, people found to have other eye health problems, or distance vision impairment, can be referred onwards for further assessment and services.

The presbyopia calculator offers several advantages over unguided trial-and-error with ready-made glasses:.

- Guidance and standardization: It provides step-by-step guidance for CHWs who may be unfamiliar with the provision of near-vision glasses. Even where ready-made glasses are available on-site, unguided trial-and-error relies on the CHW’s confidence and judgement and produces no record of the outcome; the tool addresses both by standardizing the process and capturing the result.
- Efficiency: It starts from the most probable appropriate power rather than testing all available powers.
- Confirmation and safeguarding: Formalized re-testing of near visual acuity (NVA) confirms successful correction of near vision impairment (NVI) and prompts onward referral where good vision cannot be achieved, providing a safeguard so that people needing further care are reliably referred rather than lost to follow-up.
- Data collection: It enables digital data collection on NVI and correction requirements in a standardized, transferable format, supporting epidemiological monitoring and program management.

Although the Peek presbyopia calculator is standardized in terms of assessment and initial recommendation, we appreciate that different individuals have different preferences and visual requirements, and that the minimum power allowing achievement of N6 might not be most visually comfortable for the patient. The user preference step of our tool design, where a patient can choose the power they find most visually comfortable, accounts for this, and is hypothesized to empower patients to later self-select future pairs, now that they are aware of the problem and solution. A combination of options (including the Peek presbyopia calculator, WHO TAP, self-selection and others) is likely to be needed to reach the large global need.

This tool is also an example of application of large-scale healthcare program data (previously published (18)) which allowed development of the tool’s algorithm. This demonstrates how eye care programs themselves can provide useful epidemiological data. Many of us are familiar with Professor Fred Hollows’ adage: “no survey without service” (25), alluding to the obligation to provide treatment to people who are found to have problems during surveys. As data collection technology improves, we move closer to also supporting the inverse: “no service without survey”, as healthcare programs which don’t collect - and use - data, are missing an opportunity to improve future care. In contrast to other approaches, this method may offer eye health programs a clearer understanding of the potential demand for ready readers relative to other treatment options. An additional benefit is that dispensing parameters can be customized by programs, allowing prescriptions for ready readers to align with local regulations and clinical practices.

To first ensure accuracy of recommended correction, here we compared to the optometrist gold standard, however potential future steps could include comparison of the Peek presbyopia calculator to other methods of selection, for example, WHO TAP or self-selection. Other future research could additionally include the collection of downstream information such as real-world program data of its use, and results of user satisfaction. There is also potential to update the algorithm as more program data is collected, and/or to tailor it to specific populations. Moreover, the tool may be used in a range of settings to enable provision of near vision glasses for presbyopia, including community eye health programs, other healthcare programs (e.g. non-communicable disease outreach and clinics), and within non-healthcare development interventions. A wide range of channels is likely to be needed to achieve 100% near eREC, ranging from self-selection, CHW provision with tools such as the Peek presbyopia calculator and training like WHO TAP, and bespoke refractive correction from optometrists for presbyopes with co-existing more complex refractive error such as astigmatism.

## Data Availability

The de-identified data used in this study may be made available from the corresponding author on reasonable request and with data use agreements in place.
