# Supplementary material for: A novel digital tool to guide provision of near vision glasses for presbyopia correction in the community
Source: Front Ophthalmol (Lausanne). 2026 Aug 10;6:1891054. doi: 10.3389/fopht.2026.1891054 (PMC13500277; doi:10.3389/fopht.2026.1891054)
Supplement: Supplementary file 1 [file DataSheet1.pdf]

Development of a new tool to prescribe presbyopia correction

You have been selected as an individual with expertise relevant to vision testing and community eye care. The approaches to provision of presbyopia correction vary between contexts, from formal refraction and provision of personalised correction (including astigmatic correction) to easily available readymade reading glasses which can be purchased without any formal assessment. We are working to identify the optimal method to provide presbyopia correction. We would be very grateful if you could answer the following survey questions with your views about the best way to provide correction for presbyopia.

The questions in this survey cover the topic from multiple angles in detail, and some may fall outside your area of experience. If you have no preference, please feel free to leave questions unanswered where appropriate.

* Indicates required question

1. 1) Name *

2. 2) Do you have experience of vision testing in low or middle-income countries? *

Mark only one oval.

- Yes
- No

3. 3) In which context(s) do you have experience of vision testing? (please select all that apply) *

Tick all that apply.

- Clinical setting
- Research
- Community screening
- Test development
- Other:

4. 4) Do you have experience of providing near vision correction/prescription for presbyopia? *

Mark only one oval.

- Yes
- No

Section 2: Standard presbyopia testing

This section will ask you about the practice of presbyopia prescribing patterns that you currently use.

5. 5) Which type of correction do you think is most appropriate for presbyopia in general? (please select a maximum of 3 items)

Tick all that apply.

- [ ] Ready-made single vision spectacles
- [ ] Custom-made single vision spectacles
- [ ] Bifocal spectacles
- [ ] Trifocal spectacles
- [ ] Progressive addition/multifocal spectacles
- [ ] Monovision contact lenses
- [ ] Multifocal contact lenses
- [ ] Refractive surgery
- [ ] Premium IOL (e.g. EDOF)
- [ ] Eye exercises / Visual training
- [ ] Other: ____________

6. 6) How important are the following factors when selecting the power of near correction (readers) prescription for a patient?

Mark only one oval per row.

	1 (unimportant)	2	3	4	5 (very important)
**Age**	◯	◯	◯	◯	◯
**Sex**	◯	◯	◯	◯	◯
**Education level/literacy/patient cognition**	◯	◯	◯	◯	◯
**Occupation**	◯	◯	◯	◯	◯
**Lifestyle and visual demands**	◯	◯	◯	◯	◯
**Subjective complaints of near vision difficulty**	◯	◯	◯	◯	◯
**Uncorrected near visual acuity**	◯	◯	◯	◯	◯
**Distance refractive error**	◯	◯	◯	◯	◯
**Lens status (cataract/pseudophakia/aphakia)**	◯	◯	◯	◯	◯
**Other ocular comorbidities**	◯	◯	◯	◯	◯
**Diabetes or other systematic comorbidities**	◯	◯	◯	◯	◯
**Prescription complexity**	◯	◯	◯	◯	◯
**Availability of time/personnel**	◯	◯	◯	◯	◯
**Availability of technical resources, e.g. autorefractor**	◯	◯	◯	◯	◯
**Availability of distance spectacles**	◯	◯	◯	◯	◯
**Binocular vision and convergence abilities**	◯	◯	◯	◯	◯
**Amplitude of accommodation**	◯	◯	◯	◯	◯
**Previous Prescription History**	◯	◯	◯	◯	◯

7. 7) What other factors do you consider?

____

8. 8) Please rank the following in order of highest to lowest priority when prescribing.

Mark only one oval per row.

	Priority 1 (most important)	Priority 2	Priority 3	Priority 4 (least important)
**Patient satisfaction/visual outcome**	⬭	⬭	⬭	⬭
**Cost effectiveness**	⬭	⬭	⬭	⬭
**Time take per patient**	⬭	⬭	⬭	⬭
**Available powers of near correction**	⬭	⬭	⬭	⬭

9. 9) At what age do you consider giving a patient presbyopia correction? (years)

____

10. 10) How much time do you think is appropriate to spend on providing a person with correction for presbyopia?

Mark only one oval.

⬭ 1-5 minutes

⬭ 5-10 minutes

⬭ 10-15 minutes

⬭ 15-25 minutes

⬭ Over 25 minutes

11. 11) After how long do you ask your patients to return to the clinic after providing presbyopia correction?

Mark only one oval.

- 0-6 months
- 6-12 months
- 12-24 months
- 24-36 months
- Over 36 months
- As and when required
- No follow up needed
- Other:

12. 12) Is measuring the working distance a crucial factor when assessing presbyopia?

Mark only one oval.

- Yes
- No

13. 13) Is measuring the distance vision a crucial factor when providing presbyopia correction?

Mark only one oval.

- Yes: just pass/fail screening for distance visual impairment
- Yes: more detailed distance visual acuity test
- Yes: distance visual acuity + refraction
- No

14. 14) How important do you think it is to generate an accurate prescription for presbyopia?

Mark only one oval.

	1	2	3	4	5	
Unir	◯	◯	◯	◯	◯	Very important

15. 15) Which type of correction do you think is most appropriate for presbyopia in a community practice in low income settings?

Mark only one oval per row.

	1 (low priority)	2	3	4	5 (high priority)
**Ready-made single vision spectacles**	◯	◯	◯	◯	◯
**Custom-made single vision spectacles**	◯	◯	◯	◯	◯
**Bifocal lenses**	◯	◯	◯	◯	◯
**Trifocal lenses**	◯	◯	◯	◯	◯
**Progressive addition lenses**	◯	◯	◯	◯	◯
**Monovision contact lenses**	◯	◯	◯	◯	◯
**Multifocal contact lenses**	◯	◯	◯	◯	◯
**Refractive surgery**	◯	◯	◯	◯	◯
**Premium IOLs e.g. EDOF**	◯	◯	◯	◯	◯
**Eye exercises / Visual training**	◯	◯	◯	◯	◯

16. 16) Which challenges do you believe are significant with regards to presbyopia correction in a low income setting? (select all relevant items)

Tick all that apply.

- [ ] Lack of awareness among patients/the public
- [ ] Lack of awareness among healthcare/eye care workers
- [ ] Limited access
- [ ] Lack of affordability/willingness-to-pay
- [ ] Limited time
- [ ] Limited personnel
- [ ] Lack of equipment, e.g. equipment for refraction
- [ ] Other: ______________________

17. 17a) Do you find a significant difference in prescribing techniques for presbyopia between low to middle-income countries and high-income countries?

Mark only one oval.

- ( ) Yes
- ( ) No
- ( ) Don't know

18. 17b) If yes, please elaborate the differences (elaboration on above question)

19. 18a) Do you think it would be appropriate for patients to be able to select their own near correction without formal assessment? e.g. as occurs in high-income country pharmacies

Mark only one oval.

- Yes
- No
- Maybe

20. 18b) Please elaborate on your above response.

Section 3: Method of prescription

Each of us value the steps of prescription differently. Please consider which of the following steps are absolutely necessary to perform while prescribing for presbyopia, in a low to medium resource community eyecare set up with high patient throughput.

21. 19) Please select how important you believe each of the following steps are when prescribing for presbyopia in a low to medium resource setting.

Mark only one oval per row.

	1 (Unimportant)	2	3	4	5 (Very important)
**Taking a detailed history**	⬭	⬭	⬭	⬭	⬭
**Identifying lifestyle and working near vision needs**	⬭	⬭	⬭	⬭	⬭
**Measuring distance visual acuity**	⬭	⬭	⬭	⬭	⬭
**Measuring uncorrected near visual acuity**	⬭	⬭	⬭	⬭	⬭
**Measuring presenting near visual acuity (if correction available)**	⬭	⬭	⬭	⬭	⬭
**Measuring working distance**	⬭	⬭	⬭	⬭	⬭
**Trialing of different lens powers**	⬭	⬭	⬭	⬭	⬭
**Measuring exact corrected near visual acuity following provision of correction**	⬭	⬭	⬭	⬭	⬭
**Measuring if they could pass near vision threshold (N6) with the prescribed power**	⬭	⬭	⬭	⬭	⬭
**Check the range of clear vision**	⬭	⬭	⬭	⬭	⬭
**Counsel the patient of the range of clear vision**	⬭	⬭	⬭	⬭	⬭

Explain the features/limitations of spectacles	⬭	⬭	⬭	⬭	⬭

Explain the features/limitations of spectacles

22. **Persons age**
Lens power
35 to 45 +1.00
45 to 50 +1.50
50 to 55 +2.00
Over 55 +2.50 or higher

20a) The above values were suggested by du Toit (2006) as near add powers for people with presbyopia according to their age. Do you agree with these ranges?

Mark only one oval.

	1	2	3	4	5	
Stro	⬭	⬭	⬭	⬭	⬭	Strongly Agree

23. 20b) Please elaborate on your above response.

24. 21) In a low-income country community setting, do you think patients identified to have cataract should wait until after their cataract operation for presbyopia correction?

Mark only one oval.

- Yes (if both eyes have cataract)
- Yes (if at least one eye has cataract)
- No

25. 22) How long after cataract surgery do you think patients should wait to obtain presbyopia correction?

Mark only one oval.

- Minimum 6 weeks after last scheduled operation
- Less than 6 weeks after last scheduled operation
- No waiting time required

Section 4: Future of presbyopia prescription

If you would like to see some existing apps which are available, for consideration when answering the following questions, please view this short video (2min30).

26. 23a) Do you think that the way presbyopia correction is provided needs to be updated?

Mark only one oval.

- Yes
- No
- Maybe

27. 23b) Please elaborate on your above response.

28. 24a) Do you believe that a digital automated tool could support a member of non-eye care personnel to provide presbyopia correction?

Mark only one oval.

- Yes
- No
- Maybe

29. 24b) Please elaborate on your above response.

30. 25) Any other comments

End of Survey

Many thanks for taking the time to answer these questions. Your contribution is appreciated.

This content is neither created nor endorsed by Google.

Google Forms
